# SARS-CoV-2 and its Multifaceted Impact on Bone Health: Mechanisms and Clinical Evidence

**DOI:** 10.1007/s11914-023-00843-1

**Published:** 2024-01-18

**Authors:** Alexander Harris, Amy Creecy, Olatundun D. Awosanya, Thomas McCune, Marie V. Ozanne, Angela J. Toepp, Melissa A. Kacena, Xian Qiao

**Affiliations:** 1 Department of Orthopaedic Surgery, Indiana University School of Medicine, Indianapolis, IN USA; 2 Department of Internal Medicine, Eastern Virginia Medical School, Norfolk, VA USA; 3 Division of Nephrology, Eastern Virginia Medical School, Norfolk, VA USA; 4 Department of Mathematics and Statistics, Mount Holyoke College, South Hadley, MA USA; 5 Enterprise Analytics, Sentara Health, Virginia Beach, VA USA; 6 Richard L. Roudebush VA Medical Center, Indianapolis, IN USA; 7 SMG Pulmonary, Critical Care, and Sleep Specialists, Norfolk, VA USA; 8 Division of Pulmonary and Critical Care Medicine, Eastern Virginia Medical School, Norfolk, VA USA

**Keywords:** SARS-CoV-2, COVID-19, Muscle, Bone, AI, Artificial intelligence, ChatGPT

## Abstract

**Purpose of Review:**

SARS-CoV-2 infection, the culprit of the COVID-19 pandemic, has been associated with significant long-term effects on various organ systems, including bone health. This review explores the current understanding of the impacts of SARS-CoV-2 infection on bone health and its potential long-term consequences.

**Recent Findings:**

As part of the post-acute sequelae of SARS-CoV-2 infection, bone health changes are affected by COVID-19 both directly and indirectly, with multiple potential mechanisms and risk factors involved. In vitro and preclinical studies suggest that SARS-CoV-2 may directly infect bone marrow cells, leading to alterations in bone structure and osteoclast numbers. The virus can also trigger a robust inflammatory response, often referred to as a "cytokine storm", which can stimulate osteoclast activity and contribute to bone loss. Clinical evidence suggests that SARS-CoV-2 may lead to hypocalcemia, altered bone turnover markers, and a high prevalence of vertebral fractures. Furthermore, disease severity has been correlated with a decrease in bone mineral density. Indirect effects of SARS-CoV-2 on bone health, mediated through muscle weakness, mechanical unloading, nutritional deficiencies, and corticosteroid use, also contribute to the long-term consequences. The interplay of concurrent conditions such as diabetes, obesity, and kidney dysfunction with SARS-CoV-2 infection further complicates the disease's impact on bone health.

**Summary:**

SARS-CoV-2 infection directly and indirectly affects bone health, leading to potential long-term consequences. This review article is part of a series of multiple manuscripts designed to determine the utility of using artificial intelligence for writing scientific reviews.

## Introduction

This is one of many articles evaluating the utility of using AI to write scientific review articles on musculoskeletal topics [1]. The first draft of this review was written by humans and ChatGPT4.0 whereby humans selected literature references, but ChatGPT 4.0 completed the writing. Importantly, the article was edited and carefully checked for accuracy resulting in a final manuscript which was significantly different from the original draft. Refer to this edition’s Comment paper for more information [2]. First identified in Wuhan in December 2019 [3], the SARS-CoV-2 virus spread rapidly, leading to the COVID-19 pandemic [4], causing significant morbidity and mortality with high levels of inflammation irrespective of age, gender, race, and baseline health status [5, 6]. The clinical manifestations of infection with SARS-CoV-2 are diverse, ranging from asymptomatic infection to severe respiratory illness with diffuse alveolar damage that induces vascular tissue remodeling, multi-organ failure, and death [7–11].

The impact of SARS-CoV-2 infection can extend beyond the acute phase, with long-term effects on various organ systems [12]. As part of the post-acute sequelae of SARS-CoV-2 (PASC) infection symptoms, musculoskeletal consequences involving joint pain and muscular weakness have been reported [13–15], as well as significant concerns regarding kidney health [16, 17]. Emerging evidence suggests that SARS-CoV-2 infects various organs via angiotensin converting enzyme 2 (ACE2) receptors, present on bone cells [15], highlighting potential long-term implications on bone health.

Epidemiological data indicate that comorbidities including diabetes and hypertension are associated with higher mortality [18]. Immunological dysregulation, particularly cytokine release syndrome (CRS) [19], has been implicated in severe cases, affecting not just the respiratory system but potentially also skeletal health [15]. Recent studies have begun to elucidate the direct impact of SARS-CoV-2 on bone health, revealing a high prevalence of vertebral fractures which corresponded to higher rates of mechanical ventilation and worse prognosis [20••]. Additionally, lower bone mineral density (BMD) has been identified as a significant negative predictor for ICU admission and overall prognosis [21]. As SARS-CoV-2 infections continue to occur, understanding these long-term consequences is crucial for the comprehensive care of COVID-19 survivors. This article provides vital insights into the multifaceted ways the virus affects bone density and fracture risk, and discusses the preclinical evidence of bone loss, possible cellular mechanisms for bone loss, clinical evidence of increased fracture risk, and potential pathways that may indirectly affect bone and concurrent conditions of COVID-19 that may affect bone.

## In Vitro and Preclinical Work Indicating SARS-CoV-2 Negatively Alters Bone and Muscle

### SARS-CoV-2 Direct Infection of Bone Marrow Cells

SARS-CoV-2 has been implicated in bone abnormalities in patients, potentially due to direct infection of bone marrow cells. The ACE2 receptor is the binding site for the virus as it gains entry into the host cells [15]. It is a ubiquitous receptor that can be found throughout the body, including the bone cells that regulate bone turnover [22]. Additionally, the virus possesses other means of regulating bone remodeling, independent of the ACE-2 receptor.

Potential direct effects of the virus involve osteoblast and osteoclast activity through the ACE2/Ang-(1-7)/MasR axis [22], and previous research during the SARS pandemic indicated the presence of ACE2 on CD14+ monocytes, a precursor to osteoclasts [23]. SARS-CoV also possesses the accessory protein, 3a/X1, which enhances osteoclast differentiation and induces tumor necrosis factor alpha (TNF-α) in human cell lines and receptor activator of nuclear factor kappa beta ligand (RANKL) in murine cell lines, suggesting a role in bone abnormalities [23].

Another study found the SARS-CoV-2 Spike-protein upregulates senescence-associated secretory phenotype (SASP) markers in bone marrow derived macrophages (BMDMs) [24]. The Spike-protein influences the expression of SASP-related inflammatory cytokines and cathepsins in mouse macrophages differentiated by macrophage-colony stimulating factor (M-CSF) and interleukin (IL) 34-differentiated BMDMs, showing they can be infected [24]. Collectively, these studies illustrate multiple potential mechanisms through which SARS-CoV-2 can contribute to bone abnormalities.

### Alterations to Bone Structure and Osteoclast Numbers in Vivo

Recent studies have begun to elucidate the potential effects of SARS-CoV-2 infection on bone health, with a particular focus on alterations to bone structure and osteoclast numbers. The use of animal models has been instrumental in understanding the pathogenesis, transmission, and host response to SARS-CoV-2 infection [25]. Various models, including those using mice and golden Syrian hamsters, have been developed to mimic the human response to the virus [26–28]. One such model utilized a replication-deficient adenovirus to deliver human ACE2 into mice, thereby making them susceptible to SARS-CoV-2 and enabling the study of viral pathogenesis [29]. Another study compared binding affinities of SARS-CoV-2 to ACE2 across different species, suggesting a link between binding affinity and susceptibility to infection, and showing strong binding affinity in humans [30].

In a study published in 2022, we demonstrated significant reductions in several bone parameters in transgenic mice infected with SARS-CoV-2, including asymptomatic, moderately affected, and severely affected mice, within two weeks of infection [31••]. We reported a 24.4% decrease in trabecular bone volume fraction, a 19.0% decrease in trabecular number, and a 9.8% increase in trabecular separation. Correspondingly, there was also a robust 64% increase in osteoclast number, a 27% increase in osteoclast surface, and a 38% increase in osteoclasts per bone surface. Given that asymptomatic mice equally demonstrated a decrease in bone volume and an increase in osteoclast number, the mechanism of bone loss was not due to a lack of mechanical loading based on disease severity. Another study using the same transgenic mouse model also found that SARS-CoV-2 infection led to acute bone loss and increased osteoclast numbers, in addition to thinner growth plates [32•]. Similarly, a study by Qiao et al. [33••] using golden Syrian hamsters found that SARS-CoV-2 infection led to increased osteoclastogenesis, resulting in decreased trabecular bone in both long bones and the axial skeleton.

A separate study by Gao et al shows SARS-CoV-2 can gain entry outside of the ACE2 receptor pathway. In their study, the virus utilized the neuropilin-1 receptors (NRP1) to infect the BMDM [34]. The alternative pathway also led to differing effects of the virus on osteoclastic activity, as it was decreased rather than increased as previously found in the transgenic mouse model that incorporated the human ACE2 (hACE2) receptor into the mice [32•]. Also of interest, and potential etiology of incongruent findings, is the use of different viral strains in the inoculum, USA-WA1/2020 and the B.1.351 strain in the hACE2 vs NRP1 models, respectively.

### Direct Effects of SARS-CoV-2 on Muscle

Myalgias and generalized weakness are common symptoms among COVID-19 patients and such symptoms could be indicative of muscular involvement. Genetic analysis of muscle tissues revealed that several cell types in human skeletal muscle express transmembrane protease, serine 2 (TMPRSS2), a protein known to facilitate SARS-CoV-2 entry into cells [13]. However, only a subset of these cells, specifically smooth muscle cells and pericytes, express ACE2 receptors. This expression pattern suggests a potential for direct viral infection in these muscle tissues. However, no direct evidence of viral infection in muscle fibers, as indicated by the absence of positive results in immunohistochemistry and electron microscopy studies, has been found [35]. It appears that the muscle inflammation observed may not be due to a direct viral infection, but rather due to an immune-mediated response to the virus. Such severe musculoskeletal manifestations necessitate further exploration to ascertain whether they result from direct viral invasion or indirect systemic responses like cytokine release, hyperlactemia, or hypoxia [36]. The indirect effects of SARS-CoV-2 infection in muscle will be discussed in more detail later.

Preclinical models and in vitro work with SARS-CoV-2 have provided valuable insights into the potential impact of the virus on bone and muscle health. These studies indicate that SARS-CoV-2 infection can lead to significant alterations in bone structure and most studies suggest it can increase osteoclast numbers, potentially contributing to bone loss. So far, there has not been direct evidence that SAR-CoV-2 infects skeletal muscle, indicating the muscular symptoms accompanying infection occur indirectly. However, further research is needed to fully understand the mechanisms underlying these effects and to develop effective therapeutic strategies.

## Cellular Mechanisms for Changes in Bone with SARS-CoV-2 Infection

### Inflammation and SARS-CoV-2: Implications for Bone Health

Age and acute inflammatory conditions can induce changes in collagen quality, which has a downstream effect on bone health [37]. Additionally, underlying chronic inflammation states such as obesity and diabetes can exacerbate the acute inflammatory state, further impacting bone health [38]. Elevations in various inflammatory markers and pro-inflammatory chemokines have been well documented in SARS-CoV-2 infection and corresponded with disease severity. An early meta-analysis found elevations in the inflammatory markers c-reactive protein, IL-6, and TNF-α were elements of poor prognosis [8]. SARS-CoV-2 infection increases inflammatory markers with known direct and indirect effects on bone health, including matrix metalloproteinase-1 (MMP-1) via osteoblast activity regulation [11], and the receptor activator of nuclear factor kappa B ligand (RANKL) via the receptor activator of nuclear factor kappa B (RANK)/osteoprotegerin (OPG) (RANKL/RANK/OPG) signaling pathway which is crucial in the regulation bone metabolism [39]. Of note, TNF-α is known to have synergistic effects with interferon gamma (IFN-γ) to induce a cytokine storm [40].

### NLRP3 Inflammasome: A Key Player in Bone Health and SARS-CoV-2 Infection

The nucleotide-binding oligomerization domain leucine rich repeats-containing receptors family, pyrin domain containing 3 (NLRP3) inflammasome, a component of the innate immune system, significantly influences bone health and disease [41, 42], and its role is increasingly relevant in the context of SARS-CoV-2 infection. Indeed, the inflammasome is highly activated in rheumatoid arthritis [43], a condition that impacts bone health, and its inhibition has been shown to mitigate joint inflammation and bone destruction [44, 45]. NLRP3 inflammasome activation is a known common pathway in various inflammatory conditions, including its role in the pathogenesis of hydroxyapatite-related conditions [46]. It impacts osteoporosis and skeletal development by altering osteoblast and osteoclast differentiation, with overexpression linked to excessive bone resorption and inadequate osteogenesis [47, 48]. The NLRP3 inflammasome activates cytokines IL-1β and IL-18, which can increase osteoclast differentiation [48, 49]. In acute SARS-CoV-2 infection, the ACE2 receptor interaction with SARS-CoV-2 Spike protein activates the NLRP3 inflammasome in very small embryonic-like stem cells (VSELs) and hematopoietic stem cells (HSCs), potentially leading to cell death by pyroptosis [50]. Furthermore, patients with severe SARS-CoV-2 infection exhibit increased NLRP3 inflammasome activation, associated with higher levels of IL-18 and active caspase-1 [51•]. Drynaria fortunei, a traditional medicine, has shown promise in attenuating NLRP3 inflammasome-mediated inflammation and improving BMD in postmenopausal osteoporosis patients [52]. Thus, the NLRP3 inflammasome's role in bone health and SARS-CoV-2 infection pathogenesis offers promising avenues for future research and potential therapeutic interventions.

### Th17 Cells: Intersecting Bone Homeostasis and SARS-CoV-2 Infection Pathogenesis

T-helper 17 cells (Th17 cells), a subset of CD4+ T cells, are pivotal in bone homeostasis and SARS-CoV-2 infection immune responses. Th17 cells produce IL-17, a stimulator of osteoclastogenesis, and express RANKL, a key mediator of bone homeostasis [53, 54]. Th17 cells are integral to fracture healing; with one study showing their egress from the gut and homing to the callus essential for bone repair [55]. In SARS-CoV-2 infection, Th17 cells contribute to the pathogenesis of the cytokine storm [56], which is known to contribute to bone loss, and elevated levels of IL-17 have been observed in mild SARS-CoV-2 cases, further implicating Th17 cells in bone dysregulation [57]. Moreover, an imbalance between Th17 and regulatory T cells is known to contribute to bone related diseases [58], providing another mechanism through which SARS-CoV-2 infection can impact bone. Understanding the interplay between Th17 cells, bone metabolism, and viral pathogenesis may provide insights into therapeutic strategies for bone disorders and SARS-CoV-2 infection.

### Hypoxia and SARS-CoV-2 Infection: Cellular Mechanisms and Implications for Bone Health

In the critical stages of SARS-CoV-2 infection, it can cause hypoxia [59, 60], a state of low oxygen delivery to tissues. Hypoxia can influence bone remodeling by tipping the balance towards bone resorption [61]. Xiang et al. [59] linked hypoxia to vascular endothelial injury and thrombotic inflammation, which are key factors in SARS-CoV-2 infection progression. Thromboembolic events, even in recovering patients, can exacerbate hypoxia. The study also showed that obesity, a risk factor for these complications, can exacerbate pulmonary dysfunction and lead to hypoxemia and thrombotic inflammation.

Hypoxia can promote bone resorption by increasing osteoclasts, both in terms of number and volume [62]. It also decreases osteoblast function through acidosis, which can occur both locally and systemically, inhibiting bone mineralization [63]. Erythropoietin (EPO), a glycoprotein often upregulated in hypoxic conditions, and its receptor EpoR have been shown to modulate both osteoblast and osteoclast activity, adding another layer of complexity to the hypoxia-bone health relationship [64]. While it can stimulate bone formation and angiogenesis [65, 66], high levels of EPO can suppress bone formation and lead to increased bone resorption via the EPO/EpoR signaling pathway [64, 67]. Anemia, a condition often related to hypoxia, is associated with lower whole-body BMD and could be a result of systemic inflammation, further affecting bone health [68]. Chronic obstructive pulmonary disease (COPD), a condition often accompanied by hypoxia, has been linked to abnormal BMD, further emphasizing the multifaceted impact of hypoxia on bone health [69]. Lastly, hypoxia has been directly linked to bone metabolism, leading to decreased BMD and an increased risk of fractures [70].

### The Role of RANK, RANKL, and OPG in Bone Changes Associated with SARS-CoV-2 Infection

The RANK/RANKL/OPG pathway is crucial in bone remodeling through a variety of mechanisms, with RANK acting as a signaling receptor to RANKL, and OPG as a decoy receptor [71]. During acute SARS-CoV-2 infection, patients with periodontitis had oral inflammation, as measured by salivary IL-6 and IL-1β [72]. This corresponded to increased hospitalizations, days in ICU, and higher supplemental oxygen requirements. Furthermore, elevated concentrations of IL-1β, RANKL, and neutrophil extracellular traps (NETs) were shown to persist even 100 days past the initial infection, all of which are key contributors to bone resorption. Another study found murine coronavirus infection to be associated with decreased OPG and increased RANKL/OPG ratio, triggered through a TNF-dependent osteoporotic phenotype in mice with SARS-like infection [73]. In the same study, a similar increase in RANKL/OPG ratio was found in the serum of acute SARS-CoV-2 infected patients. Furthermore, post-SARS-CoV-2 infection, patients showed significant decreases in BMD and increases in OPG levels [74]. Such changes in the regulation of bone remodeling during and post-SARS-CoV-2 infection could potentially lead to osteopenia or osteoporosis.

## Clinical Evidence that SARS-CoV-2 Infection Results in Bone Loss

### SARS-CoV-2 and its Impact on Bone Health

Emerging research suggests that SARS-CoV-2 infection may have significant effects on bone health, potentially leading to bone loss. Two studies identified hypocalcemia, characterized by low blood calcium levels, as a prevalent condition in SARS-CoV-2 patients, and low levels correlated with increased disease severity and worse clinical outcomes [75, 76]. A third study found SARS-CoV-2 infection altered bone turnover markers, suggesting that even moderate severity of SARS-CoV-2 infection could impact bone health and potentially lead to bone loss, even without concurrent hypocalcemia [77•].

These findings increase concern that SARS-CoV-2 infection may have long-term effects on bone health, underscoring the importance of evaluating patients’ bone turnover markers and fracture risk post-recovery.

### Vertebral Fractures in SARS-CoV-2 Patients: A Significant Concern

The prevalence and impact of vertebral fractures (VFs), an often overlooked diagnosis in SARS-CoV-2 patients, has emerged as a significant health concern. Detection of VFs ranged between 22% and 36% of SARS-CoV-2 patients who presented to the emergency department [20]. The same study showed the presence of VFs was an independent predictor of the need for noninvasive mechanical ventilation and while the presence of VF was not statistically significant for mortality, the patients with severe fracture did have a statistically significant difference in mortality, indicating VFs as a marker of fragility and poor prognosis. The authors of the paper concluded it would be beneficial that all patients who present with symptoms of SARS-CoV-2 infection and undergo a chest x-ray receive a morphometric thoracic vertebral evaluation. Another study concluded VFs only significantly increased mortality when there were multiple VFs, but significantly increased mortality risk in non-SARS-CoV-2 patients with both single and multiple VFs [78]. The authors highlighted the importance of diagnosing fragility fracture in SARS-CoV-2 patients and non-SARS-CoV-2 patients to raise awareness of treatment with vitamin D and anti-osteoporosis drugs. The high prevalence and potential impact of VFs during infection accentuate the need for increased awareness and proactive management of this complication in both SARS-CoV-2 and non-SARS-CoV-2 patients.

### The Impact of SARS-CoV-2 Infection on Bone Mineral Density and its Correlation with Disease Severity

Another concerning complication of SARS-CoV-2 infection is the significant decrease in BMD, which is correlated with the severity of the disease, leading to concerns for the long-term health of survivors [21, 79–81]. Patients with SARS-CoV-2 who have a BMD below 80 mg/ml were found to have over a 75% increased likelihood of needing intensive care treatment, making BMD a valuable predictor for treatment [79]. This risk is further compounded by factors such as the patient's age, physical condition, comorbidities, physical activity, body composition, and lifestyle [21, 79]. Moreover, a lower BMD, defined as ≤100 HU, was significantly associated with higher rates of mortality, ICU admission, and mechanical ventilation in SARS-CoV-2 patients [21]. The link between clinical categorization and reduced BMD underscores the significance of BMD as a key standalone indicator of mortality, which can be readily assessed from the chest CT scans of SARS-CoV-2 patients. [21].

Expanding upon these findings, recent research by Federica Buccino et al. [82•] has delved into the micro-architectural alterations in bone due to SARS-CoV-2 infection. The study found that the virus induces changes at the lacunar level of bone architecture, akin to those observed in osteoporotic conditions. This revelation adds a new dimension to our understanding, suggesting that the impact of SARS-CoV-2 infection on bone health may be more intricate and pervasive than previously thought. The study also emphasized the importance of long-term monitoring of bone mass and strength in SARS-CoV-2 survivors, given these micro-scale alterations.

The negative impact of SARS-CoV-2 infection on BMD is further evidenced by a significant decrease in BMD of the lumbar region and femur at 9 months as compared to baseline in patients who contracted SARS-CoV-2 [80]. In fact, the same study found that this response was graded, with the most severe SARS-CoV-2 patients having the highest loss in BMD. Corticosteroid, one of few proven medications for treating SARS-CoV-2 infection [83], also adversely affects bone health [81]. This is especially true in elderly patients, further complicating treatment of high-risk individuals. Additionally, patients with osteopenia or osteoporosis before SARS-CoV-2 infection had significant detriments to their bone health, including decreased BMD, highlighting the importance of outpatient osteoporosis treatment prior to hospitalization. [81].

Adding to the body of evidence, a study by Al-Azzawi and Mohammed found that SARS-CoV-2 infection significantly affects bone remodeling, potentially leading to conditions such as osteopenia or osteoporosis [74]. The researchers found a statistically significant difference in the mean OPG level and BMD, measured by DEXA scan, between post-SARS-CoV-2 patients and non-SARS-CoV-2 subjects which the researchers matched according to age and BMI range. This suggests that SARS-CoV-2 may disrupt the balance of bone homeostasis, leading to a decrease in BMD. Therefore, monitoring OPG levels in the serum could be helpful in predicting low BMD in post-SARS-CoV-2 patients. As the number of patients with post-acute sequelae of SARS-CoV-2 infection (PASC patients) continues to increase, it may be crucial to monitor their bone health status closely and consider osteoporosis therapies for those who required long-term corticosteroid treatment.

## Indirect Effects of SARS-CoV-2 Infection on Bone

### Muscle Weakness, Mechanical Unloading, and the Indirect Effects of SARS-CoV-2 Infection on Bone Health

Muscle breakdown, especially during critical illnesses, has systemic implications. ICU stays often result in muscle weakness due to both atrophy and impaired contractile function [84, 85]. This is intertwined with mitochondrial dysfunction, suggesting that critical illnesses might mirror acquired mitochondrial disorders [85]. Systemic inflammation, prevalent in these conditions, intensifies muscle degradation, with cytokines both promoting protein breakdown and hampering muscle regeneration [86–88]. One study observed that in 146 COVID-19 patients who developed rhabdomyolysis, approximately 30% died and 40% developed AKI, indicating the systemic effects of muscle breakdown can result in severe consequences for COVID-19 patients [89].

As previous studies have shown, there is a complex interplay between muscle and bone, with secreted factors from muscle potentially influencing bone health, and vice versa [90]. Furthermore, daily physical activity requiring muscle strain have been known to influence bone mass and architecture [90]. During the active infection, SARS-CoV-2 can cause both muscle weakness [91] and wasting [92••] in the limbs, leading to functional impairment. Immune-mediated myopathy has been observed in patients who died from SARS-CoV-2 infection, indicating a possible post-infectious, immune-mediated mechanism affecting muscle health [35]. Post-infection, SARS-CoV-2 can have a lasting effect on the interplay of muscle and bone health. In SARS-CoV-2 survivors with reduced muscle strength, there was a corresponding decrease in muscle thickness and a higher muscle stiffness score measured by ultrasound [93]. In addition to lower muscle mass, long-term consequences of SARS-CoV-2 infection include decreased exercise capacity and chronic fatigue impairing daily activities, all of which have an indirect impact on bone health [94].

Taken together, SARS-CoV-2 has both immediate and long-term effects on muscle mass and daily activities, raising concerns for additional mechanisms of bone health deterioration in PASC patients. Further research is needed to fully understand these relationships and develop effective interventions.

### Nutritional Deficiencies and their Impact on Bone Health in the Context of SARS-CoV-2 Infection

SARS-CoV-2 patients often experience nutritional challenges, including malnutrition and significant weight loss, which can impact bone health [95, 96]. Vitamin D3, or cholecalciferol, has been associated with the regulation of bone, calcium, and phosphorus metabolism, as well as the modulation of the immune system [97]. Its deficiency may lead to immune dysregulation, including impaired macrophage function and increased production of proinflammatory cytokines [98], leading to indirect effects on bone health. Zinc and selenium deficiencies have been positively associated with the incidence of SARS-CoV-2 infection and zinc deficiencies have been associated with the severity of disease, highlighting their role in immune modulation and antiviral capabilities [99]. Zinc, in particular, can regulate the RANKL/RANK/OPG pathway, a crucial axis for bone remodeling [100]. Selenium affects bone health via its regulation in the production of pro-inflammatory cytokines [101]. However, a systematic review found few studies investigating selenium supplementation and that the zinc supplementation studies did not provide evidence for efficacy in prevention or as a therapeutic for SARS-CoV-2 infection, indicating a need for further investigation and caution in utilizing supplementation as an approach for treating COVID-19 patients [102].

### Steroid Utilization and its Indirect Effects on Bone Health amid SARS-CoV-2 Infection

The COVID-19 pandemic has led to extensive steroid use due to their anti-inflammatory and immunosuppressive properties, and their use reduced mortality [83]. However, deleterious effects of steroids on bone health are significant. Steroids are known to reduce bone mass and increase bone loss, leading to osteoporosis and a higher risk of bone fracture [103]. They cause increased bone fragility by increasing matrix hypermineralization and decrease osteocyte lacunar size [104, 105].

Glucocorticoids, such as dexamethasone, directly influence osteoclast activity leading to increased bone resorption as evidenced by the upregulation of osteoclast markers such as CTR, TRAP, and cathepsin K [106]. Moreover, dexamethasone inhibits bone formation by downregulating osteocalcin, an osteoblast marker. Furthermore, corticosteroids interfere with calcium absorption and disrupt the balance of key molecules like RANKL and OPG involved in bone remodeling [107]. These studies emphasize the need for a nuanced approach in corticosteroid treatment protocols. While they are necessary, strong considerations should be afforded to the dose, duration, and concomitant administration of medications that can possibly mitigate the deleterious effects.

## Concurrent Conditions and Severity of SARS-CoV-2 Infection

### The Interplay of Diabetes, Obesity, and SARS-CoV-2 Infection Severity

The interplay between diabetes, obesity, and SARS-CoV-2 infection is complex, involving metabolic dysregulation, chronic inflammation, and impaired immune response [108]. Diabetes and obesity, known to impair immune function, have been identified as risk factors for severe SARS-CoV-2 infection outcomes, including increased hospitalization rates and mortality [5, 109]. These conditions are characterized by metabolic dysregulation and chronic inflammation, which can exacerbate the severity of SARS-CoV-2 infection [38, 109]. In addition to increasing the prevalence of fractures [110], diabetes may also affect the efficacy of therapies against SARS-CoV-2 infection [111] and cause increased inflammation and a protracted course of infection and recovery. For SARS-CoV-2 infection survivors, a systematic review found a 64% greater risk of new onset diabetes when comparing patients with SARS-CoV-2 infection to patients without it [112]. These studies bring to light the necessity for vigilant diabetes management and risk assessment in SARS-CoV-2 patients. Understanding these interactions is crucial for managing SARS-CoV-2 during and after an active infection in these high-risk populations.

### Acute Kidney Injury and Chronic Kidney Disease in SARS-CoV-2 Infection

SARS-CoV-2 infection has been associated with significant complications of acute kidney injury (AKI) and chronic kidney disease (CKD) [113]. In fact, one study found that of 5449 patients admitted for SARS-CoV-2 infection in 13 New York hospitals, 36.6% developed AKI [114]. This number increases to 89.7% when examining only patients with respiratory failure who were placed on mechanical ventilation. The transition from AKI to CKD, termed acute kidney disease (AKD), involves inflammation and fibrosis mediated by maladaptive repair [115]. CKD is also known to result in poor bone quality and a higher incidence of fracture [116].

Individuals who have recovered from SARS-CoV-2 and experienced AKI show an increased rate of significant cardiovascular and kidney complications, especially if their recovery from AKI took longer or was extended [117]. Moreover, AKI requiring temporary dialysis has been shown to independently increase the long-term risk of bone fractures [118••]. This is particularly concerning given the already elevated risk of fractures in CKD patients due to mineral and bone disorders (CKD-MBD) [119]. CKD-MBD is a syndrome involving imbalances in calcium, phosphate, parathyroid hormone (PTH), and vitamin D metabolism [120]. The role of fibroblast growth factor 23 (FGF-23) in regulating phosphate homeostasis and vitamin D metabolism is also noteworthy, especially in the context of CKD [121, 122].

Recent meta-analyses have not found a significant difference in inactive vitamin D levels between AKI and non-AKI patients; however, active vitamin D levels were significantly lower in AKI patients [123]. Secondary hyperparathyroidism (sHPT), often seen in CKD, is linked to adverse outcomes like kidney disease progression and cardiovascular events [124]. Effective management of sHPT and CKD-MBD is crucial, given the associated risks of hypercalcemia and hyperphosphatemia.

These interconnected syndromes present significant complications for bone health in SARS-CoV-2 patients, necessitating further research for effective treatments and preventive measures [125]. Additionally, systemic activation of activin A, a member of the transforming growth factor family, seems to be implicated in CKD-MBD [126] The complexities of vitamin D deficiencies and sHPT in CKD patients further underscore the need for a multidisciplinary approach in managing these patients [125, 126].

## Conclusion

**Fig. 1:**
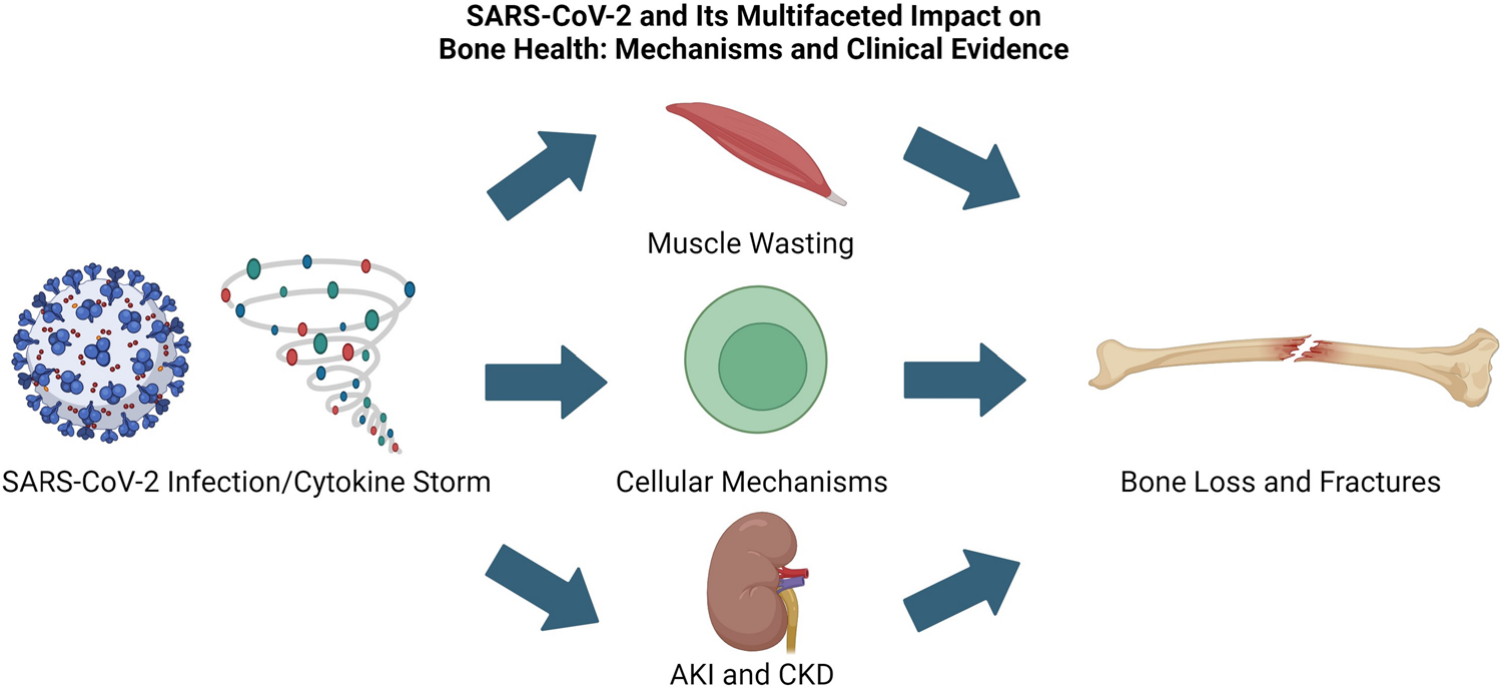
Overview of mechanisms through which SARS-CoV-2 can cause bone loss

The COVID-19 pandemic has significant long-term effects on various organ systems, including bone health. The disease has been associated with hypocalcemia, altered bone turnover markers, and a high prevalence of VFs. The severity of the disease correlates with a decrease in BMD. As shown in Figure 1, there are multiple potential mechanisms of SARS-CoV-2's impact on bone metabolism, including direct infection of bone marrow cells, inflammation, and activation of the NLRP3 inflammasome. Indirect effects, such as muscle weakness, nutritional deficiencies, corticosteroid use, and decreased physical activity in PASC patients also impact bone health. The interplay of diabetes, obesity, and SARS-CoV-2 infection severity further complicates the disease's impact on bone health. Additional complications to bone result from kidney dysfunctions such as AKI and CKD. Comprehensive research is needed to understand these effects, develop effective interventions to protect long-term bone health, and tailor clinical approaches to mitigate these long-lasting effects on patients' health.
